# Functional characterization of NRT1/PTR FAMILY transporters: looking for a needle in a haystack

**DOI:** 10.1111/nph.70730

**Published:** 2025-11-23

**Authors:** Laura Morales de Los Ríos, The Dan Pham, Thibaut Perez, Myriam Ben Amar, Claire Corratgé‐Faillie, François Barbier, Benoit Lacombe

**Affiliations:** ^1^ Institute for Plant Sciences of Montpellier, IPSIM Université de Montpellier/CNRS/INRAE/Institut Agro Montpellier 2 Place Pierre Viala Montpellier 34060 France

**Keywords:** development, hormones, nutrients, transporter, *Xenopus* oocytes, yeast

## Abstract

NRT1/PTR FAMILY (NPF) transporters play crucial roles in plant physiology and development due to their involvement in nitrogen nutrition and their ability to transport multiple signaling molecules and metabolites. Whereas most eukaryotic and prokaryotic NPF orthologs are peptide transporters, most flowering plant NPF transport other substrates such as nitrate as well as a wide range of structurally unrelated molecules like amino acids, potassium, chloride, glucosinolates, alkaloids, nicotianamine, sugars, or glycerate. Some NPF have also been reported to transport hormones, such as auxin, abscisic acid, gibberellins, or jasmonate. Strikingly, several NPF were shown to transport more than one of these molecules. This multispecificity places NPF in a central position in the integration of plant signals. In addition, different plant NPF were reported to have a signaling activity that is independent of their transport activity, indicating a direct role in molecule sensing. Identification of NPF substrates is not trivial and requires specific heterologous expression systems such as *Xenopus* oocytes or yeast to be functionally characterized. The aim of this review is to highlight the techniques used for NPF characterization, provide a comprehensive overview of their different substrates, and to speculate on the role of NPF during plant evolution.

**Table:** 

	Content	
	Summary	1124
I.	Introduction	1124
II.	Characterization methods of NPF transport activity	1125
III.	Substrate transport selectivity of NPF	1132
IV.	Evolution of NPF in plants	1138
V.	Conclusion, recommendations, and perspectives	1139
	Acknowledgments	1140
	References	1140

## I. Introduction

Living organisms need selective transporters to maintain gradients across membranes in order to survive. This enables them, for example, to import nutrients from their environment and transport signaling molecules. To achieve this, superfamilies of transmembrane proteins enabling molecule transport have evolved in all living organisms. Among them, a family of proteins initially functionally characterized as dipeptide transporters is found in the three domains of life. This family is referred to as PROTON‐COUPLED OLIGOPEPTIDE TRANSPORTER (POT), PEPTIDE TRANSPORTER (PepT/PTR), or SOLUTE CARRIER 15 (SLC15) (Hauser *et al*., 2001; Daniel, 2004; Newstead *et al*., 2011). POT/PTR/SLC15 proteins found in nonplant organisms primarily specialize in H^+^/peptide cotransport and participate in nitrogen nutrition. In plants, this transporter family is known as NITRATE TRANSPORT 1/PTR FAMILY (NPF) (Léran *et al*., 2014). Strikingly, in flowering plants, where most of the NPF have been characterized, the members of this family have greatly diversified in number and substrate specificity, making them stand out compared with the rest of the POT/PTR/SLC15 found in other organisms. In 2014, the study of phylogenetic relationships between the plant members of the NPF family allowed the identification of eight unambiguous clades (Léran *et al*., 2014) which was used as a bedrock for the nomenclature of these transporters.

With the cloning of AtNPF6.3/NRT1.1/CHL1 in 1993, Tsay *et al*. (1993) demonstrated the ability of this NPF to cotransport protons and nitrate (NO_3_^−^). In the following years, additional nitrate transporters within this family were identified in various plant species (Supporting Information Table S1). Histidine and dipeptides were the next types of substrates discovered for plant NPF (Frommer *et al*., 1994; Rentsch *et al*., 1995; Chiang *et al*., 2004; Dietrich *et al*., 2004). Subsequently, researchers revealed a wide range of structurally unrelated molecules as substrates of various NPF transporters, including nitrite (NO_2_^−^), chloride (Cl^−^), potassium (K^+^), glucosinolates (GSLs), alkaloids, histidine, nicotianamine (NA), sucrose, glucose, glycerate, and several plant hormones such as auxin (IAA and its precursor IBA), abscisic acid (ABA), jasmonates (JAs), and gibberellins (GAs) (Corratgé‐Faillie & Lacombe, 2017; Y.‐Y. Wang *et al*., 2018; Kanstrup & Nour‐Eldin, 2022). Some of these transport proteins have been shown to be proton‐coupled transporters (cotransporters) in the same (symport) or inverse (antiport) direction. For some NPF substrates (GSLs or dipeptides), transport activity appears relatively confined to distinct subclades (NPF2b and NPF8, respectively) with a high degree of amino acid identity. By contrast, for other substrates like GAs or nitrate, there is no apparent phylogenetic clustering within specific subclades. Moreover, some NPF proteins can transport two or more of these substrates (Tables 1, S1), making NPF transporters critical proteins in the interactions between different signaling pathways.

**Table 1:** NRT1/PTR FAMILY (NPF) in *Arabidopsis thaliana*.

Locus	NPF name	Other name	Nitrate transport	Other substrates	Characterization system	Cellular localization	Tissue expression	References
At3g16180	NPF1.1	NRT1.11	Yes (*K*_m_ 7.2 mM)	GA, JA‐Ile	Oocytes and yeast	PM	Companion cells of the major veins leaves	Hsu & Tsay (2013); Chiba *et al*. (2015)
At1g52190	NPF1.2	NRT1.12	Yes (*K*_m_ 9.2 mM)	GA, JA‐Ile	Oocytes and yeast	PM	Companion cells of the major veins leaves	Hsu & Tsay (2013); Chiba *et al*. (2015)
At5g11570	NPF1.3		Yes (*K*_m_ 2.5–10 mM)		Oocytes and yeast	PM		Chen & Ho (2022)
At3g45720	NPF2.1			GA	Oocytes			Wulff *et al*. (2019)
At3g45690	NPF2.2							
At3g45680	NPF2.3	NAXT2	Yes	GA	Bacteria, Oocytes, and yeast	PM	Root pericycle cells (mature roots)	Chiba *et al*. (2015); Taochy *et al*. (2015); Wulff *et al*. (2019)
At3g45700	NPF2.4		Yes	Cl^−^, GA, ± JA‐Ile	Oocytes and yeast	PM	Root stelar cells (also in leaves, flowers, and siliques)	Chiba *et al*. (2015); Li *et al*. (2016); Wulff *et al*. (2019)
At3g45710	NPF2.5			Cl^−^, GA, ABA	Oocytes and yeast	PM	Root cortical cells	Chiba *et al*. (2015); B. Li *et al*. (2017); Wulff *et al*. (2019)
At3g45660	NPF2.6			GA, ± JA‐Ile	Yeast			Chiba *et al*. (2015)
At3g45650	NPF2.7	NAXT1	Yes (*K*_m_ 5 mM)	GA, ± JA‐Ile	Liposomes, oocytes, and yeast	PM	Root cortical cells (mature roots)	Segonzac *et al*. (2007); Chiba *et al*. (2015); Wulff *et al*. (2019)
At5g28470	NPF2.8	FST1	No influx	Flavonoids	Bacteria	PM	Anther (tapetum)	Léran *et al*. (2015a,b); Grunewald *et al*. (2020)
At1g18880	NPF2.9	NRT1.9/GTR3	Yes (*K*_m_ 7 mM)	GA, GSL	Oocytes	PM	Root phloem (companion cells)	Wang & Tsay (2011); Jørgensen *et al*. (2017); Wulff *et al*. (2019); Kanstrup *et al*. (2023)
At3g47960	NPF2.10	GTR1	Yes	GA, GSL, JA, ± JA‐Ile	Oocytes and yeast	PM	Leaves (adjacent mesophyll cells)	Nour‐Eldin *et al*. (2012); Andersen *et al*. (2013); Chiba *et al*. (2015); Saito *et al*. (2015); Ishimaru *et al*. (2017); Jørgensen *et al*. (2017); Xu *et al*. (2017); Kuo *et al*. (2020); Kanstrup *et al*. (2023); Binenbaum *et al*. (2023)
At5g62680	NPF2.11	NRT1.10/GTR2	Yes	GA, GSL	Oocytes	PM	Leaves vascular tissue	Nour‐Elin *et al*. (2012); Andersen *et al*. (2013); Jørgensen *et al*. (2017); Xu *et al*. (2017); Wulff *et al*. (2019); Kanstrup *et al*. (2023)
At1g27080	NPF2.12	NRT1.6	Yes (*K*_m_ 6 mM)	ABA, GA	Oocytes and yeast	PM	Vascular tissue of siliques	Almagro *et al*. (2008); Chiba *et al*. (2015); Saito *et al*. (2015); Wulff *et al*. (2019); Binenbaum *et al*. (2023)
At1g69870	NPF2.13	NRT1.7	Yes (*K*_m_ 2.8 mM)	ABA, GA, GSL, ± JA‐Ile	Oocytes and yeast	PM	Older leaves phloem	Fan *et al*. (2009); Chiba *et al*. (2015); Jørgensen *et al*. (2017); Wulff *et al*. (2019); Binenbaum *et al*. (2023)
At1g69860	NPF2.14		No influx	ABA, GA	Oocytes	TP	Root pericycle cells	Binenbaum *et al*. (2023)
At1g68570	NPF3.1	NITR	Yes	ABA, GA, JA‐Ile, nitrite	Oocytes and yeast	PM	Root endodermis cells, vascular tissue, and leaves	Sugiura *et al*. (2007); Pike *et al*. (2014); Chiba *et al*. (2015); David *et al*. (2016); Tal *et al*. (2016); Binenbaum *et al*. (2023)
At3g25260	NPF4.1	AIT3		ABA, GA	Insect cells and yeast	PM	Endosperm	Kanno *et al*. (2012); Kanno *et al*. (2013); Sirlin‐Josserand *et al*. (2024)
At3g25280	NPF4.2	AIT4		ABA	Oocytes and yeast			Kanno *et al*. (2012); Léran *et al*. (2020)
At1g59740	NPF4.3	NRT1.14	No influx		Oocytes			Kanno *et al*. (2012); Léran *et al*. (2015a,b); Léran *et al*. (2020)
At1g33440	NPF4.4	NRT1.13	No influx, transceptor?		Oocytes and yeast	PM	Xylem parenchyma cells	Kanno *et al*. (2012); Léran *et al*. (2020); Chen *et al*. (2021a)
At1g27040	NPF4.5	AIT2	No influx	ABA	Oocytes and yeast			Kanno *et al*. (2012); Léran *et al*. (2015a,b); Léran *et al*. (2020)
At1g69850	NPF4.6	NRT1.2/AIT1	Yes (*K*_M_ 5.9 mM)	ABA, K^+^, Na^+^, Li^+^	Insect cells, oocytes, and yeast	PM	Root hair and root epidermis	Huang *et al*. (1999); Kanno *et al*. (2013); Léran *et al*. (2020); Shimizu *et al*. (2021); Liu *et al*. (2025)
At5g62730	NPF4.7			ABA	Oocytes			Léran *et al*. (2020)
At2g40460	NPF5.1		No influx	ABA, GA	Oocytes and yeast		Seed coat, leaves, vascular tissues, and mesophyll	Chiba *et al*. (2015); Léran *et al*. (2015a,b); Shimizu *et al*. (2021); Shimizu *et al*. (2022)
At5g46050	NPF5.2	PTR3		ABA, dipeptides, GA	Yeast		Roots, leaves, and seeds	Karim *et al*. (2005); Karim *et al*. (2007); Chiba *et al*. (2015)
At5g46040	NPF5.3		No influx	ABA	Oocytes and yeast			Chiba *et al*. (2015); Léran *et al*. (2015a,b)
At3g54450	NPF5.4		No influx					Léran *et al*. (2015a,b)
At2g38100	NPF5.5		Yes		Oocytes		Embryo	Léran *et al*. (2015a,b)
At2g37900	NPF5.6			GA	Yeast			Chiba *et al*. (2015)
At3g53960	NPF5.7		No influx	ABA, GA, JA‐Ile	Yeast			Chiba *et al*. (2015); Léran *et al*. (2015a,b)
At5g14940	NPF5.8	NAET1	Yes	Nicotianamine	Oocytes and yeast	SV	Vascular bundle (roots and leaves)	Chao *et al*. (2021); Chen *et al*. (2021b)
At3g01350	NPF5.9	NAET2	Yes	Nicotianamine	Oocytes and yeast	SV	Vascular bundle (roots and leaves)	Chao *et al*. (2021); Chen *et al*. (2021b)
At1g22540	NPF5.10		Yes (*K*_M_ 1.902 mM)		Oocytes	TP	Vascular tissue (roots and leaves)	Lu *et al*. (2022)
At1g72130	NPF5.11		Yes (*K*_M_ 2.57 mM)		Oocytes	TP	Vascular stele (roots and leaves)	He *et al*. (2017)
At1g72140	NPF5.12	TOB1	Yes (*K*_M_ 4.84 mM)	IBA	Oocytes and yeast	TP and PM	Vascular stele (roots and old leaves)	He *et al*. (2017); Michniewicz *et al*. (2019); Dölfors *et al*. (2024)
At1g72125	NPF5.13	NRT1.16						
At1g72120	NPF5.14	NRT1.15	Yes (*K*_M_ 5.37 mM)		Oocytes	TP	Vascular tissue (roots and leaves)	Lu *et al*. (2022)
At1g22570	NPF5.15							
At1g22550	NPF5.16		Yes (*K*_M_ 2.91 mM)		Oocytes	TP	Vascular stele (roots and young leaves)	He *et al*. (2017); Lu *et al*. (2022)
At5g13400	NPF6.1							
At2g26690	NPF6.2	NRT1.4	Yes		Oocytes and yeast	PM	Petiole	Chiu *et al*. (2004); Morales de los Ríos *et al*. (2021)
At1g12110	NPF6.3	NRT1.1	Yes, transceptor	Cl^−^, Chlorate, IAA	Liposomes, oocytes, and yeast	PM	Root epidermal cells and guard cells	Tsay *et al*. (1993); Guo *et al*. (2001); Ho *et al*. (2009); Krouk *et al*. (2010); Parker & Newstead (2014)
At3g21670	NPF6.4	NRT1.3/NTP3				PM	Leaves mesophyll cells and stem cortex cells	Tong *et al*. (2016)
At5g19640	NPF7.1			IAA		PM	Flowers (anther and pollen)	Babst *et al*. (2019)
At4g21680	NPF7.2	NRT1.8	Yes		Oocytes	PM	Xylem parenchyma cells, flowers, and siliques	Tsay *et al*. (2007); Li *et al*. (2010)
At1g32450	NPF7.3	NRT1.5	Yes	IBA, K^+^	Oocytes and yeast	PM	Root pericycle cells	Lin *et al*. (2008); Li *et al*. (2017b); Watanabe *et al*. (2020)
At3g54140	NPF8.1	PTR1	No influx	Di and tripeptide, dimethylarsenate	Protoplast	PM	Vascular tissue (root and leaves)	Dietrich *et al*. (2004); Komarova *et al*. (2012); Léran *et al*. (2015a,b), Tang *et al*. (2017)
At5g01180	NPF8.2	PTR5	No	Dipeptide, dimethylarsenate	Protoplast	PM	Flower (ovule and pollen)	Hammes *et al*. (2010), Tang *et al*. (2017)
At2g02040	NPF8.3	PTR2/NTR1/PTR2B	No influx	Dipetide	Oocytes and yeast	TP		Chiang *et al*. (2004); Karim *et al*. (2007); Léran *et al*. (2015a,b)
At2g02020	NPF8.4	PTR4	No	Glycerate	Oocytes and protoplast	TP	Phloem parenchyma cells (root and leaves)	Komarova *et al*. (2012); Weichert *et al*. (2012); Lin & Tsay, 2023)
At1g62200	NPF8.5	PTR6	Yes (*K*_M_ 1.56 mM)		Oocytes	TP	Old leaves and pollen	Weichert *et al*. (2012); Lu *et al*. (2022)

*K*_m_ values for nitrate transport are shown when known. ABA, abscisic acid; IAA, auxin; Cl^−^, chloride; GA, gibberellins; GSL, glucosinolates; IBA, indole‐3‐butyric acid; JA and JA‐Ile, jasmonates; Li^+^, lithium; Na^+^, sodium; K^+^, potassium. In the cellular localization column: PM, plasma membrane; TP, tonoplast; SV, secretory vesicles.

**Fig. 1:**
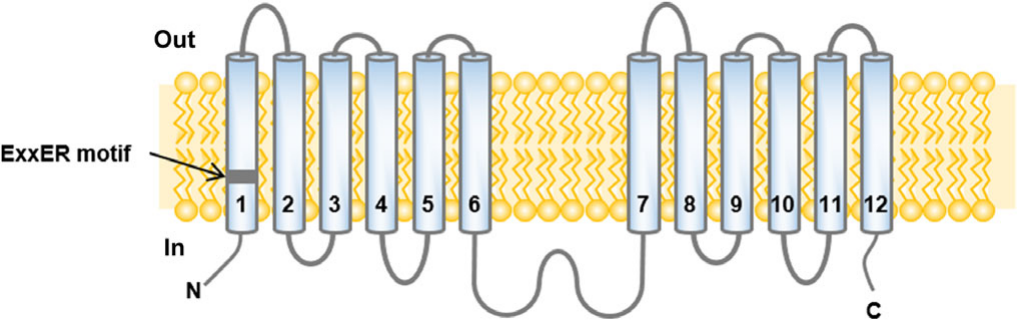
General topology of NRT1/PTR FAMILY (NPF) transporters. The numbers 1–12 indicate the transmembrane domains of NPF transporters. ExxER domain is indicated in transmembrane domain one.

Structurally, NPF possesses 12 transmembrane domains (Fig. 1), as revealed by 3D crystal structure studies of several bacterial members (Weitz *et al*., 2007; Solcan *et al*., 2012) and AtNPF6.3/NRT1.1 (Parker & Newstead, 2014; Sun *et al*., 2014). Plant NPF harbors a long hydrophilic loop positioned between transmembrane segments TM6 and TM7 (Tsay *et al*., 2007). A remarkable motif, ExxER/K (in the first transmembrane domain, Fig. 1), is conserved across many NPF members (Table S1). This motif maintains neutral residues at the xx positions across domains of life, appearing to be essential for proton‐coupling in active proton‐symporters (Solcan *et al*., 2012; Jørgensen *et al*., 2017; Newstead, 2017). Arabidopsis NPF2a transporters and the AtNPF7 clade lack this motif, suggesting potential alternative transport mechanisms. Intriguingly, some NPF7 members have an ExxER/K‐independent electrogenic proton coupling with low affinity for their substrates (Yang *et al*., 2022; Table S1), in contrast to the high affinity of the other electrogenic proton‐coupled NPFs. Understanding the molecular basis of this transport mechanism requires further electrophysiological studies.

From an agricultural point of view, the capacity of NPFs to transport different substrates makes their genetic editing a promising tool to improve crop yield, nutritional quality, and nutrient use efficiency (NUE), as well as tolerance to abiotic and biotic stresses. Studies have demonstrated the potential of certain NPF from maize and tomato to enhance NUE (Fu *et al*., 2015; Allen *et al*., 2016). In rice, allelic variation of plasma membrane–localized OsNRT1.1B/NPF6.5 was found to underpin the difference in NUE between the two subspecies *indica* and *japonica* (Koutroubas & Ntanos, 2003; Hu *et al*., 2015; Rakotoson *et al*., 2017; W. Wang *et al*., 2018). The OsNRT1.1B/NPF6.5 allele present in the *indica* subspecies has been shown to promote root microbiota diversity, consequently leading to improved nutrient uptake from the soil through an unknown mechanism (Tang *et al*., 2019; J. Zhang *et al*., 2019). Furthermore, natural variation in this gene is linked to a phenotype observed in field conditions, demonstrating its potential use as a marker for selection (Hu *et al*., 2015).

**Fig. 2:**
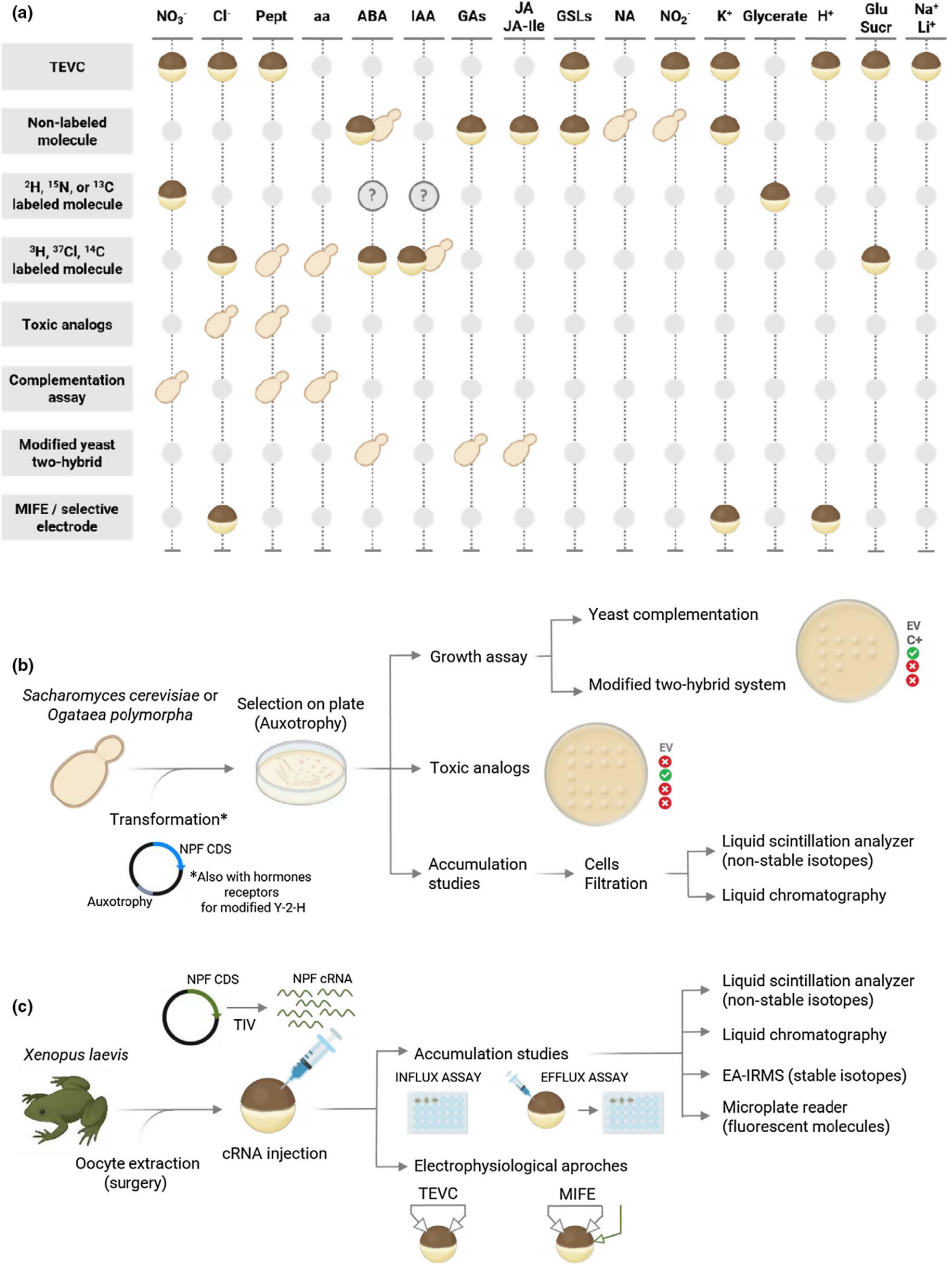
Characterization methods of NRT1/PTR FAMILY (NPF) transport activity in heterologous expression systems (oocytes and yeast). (a) Methods used to characterize NPF transport according to substrate. A question mark (?) indicates that the method could be used, although it has not yet been described in the literature. (b, c) Simplified workflow for methods using yeast or oocytes as a heterologous expression system, respectively. aa, amino acids; ABA, abscisic acid; Cl^−^, chloride; C+, positive control; CDS, coding DNA sequence; EA‐IRMS, elemental analysis and isotope ratio mass spectrometry; EV, empty vector; GAs, gibberellins; Glu/Sucr, glucose and sucrose; GSL, glucosinolates; H^+^, protons; IAA, auxin; IBA, indole‐3‐butyric acid; JA and JA‐Ile, jasmonates; K^+^, potassium; Li^+^, lithium; MIFE, microelectrode ion flux estimation system; NA, nicotianamine; Na^+^, sodium; NO_2_^−^, nitrite; NO_3_^−^, nitrate; Pept, peptides; TEVC, two‐electrode voltage clamp; Y2H, yeast two‐hybrid.

It is quite common, through transcriptomic approaches, to identify *NPF* genes that have not been functionally described in the literature, especially in less common species. Understanding the role of NPF candidates requires understanding their function, most notably by identifying their substrate(s), which is like finding a needle in a haystack. Indeed, as mentioned above, plant NPF can transport a wide range of substrates. Unfortunately, the phylogenetic classification of NPF does not correlate with their substrate specificity, making it difficult to predict the substrate of uncharacterized NPF based on their protein sequence and structure. Despite a huge effort of the scientific community to use bioinformatic tools to palliate this issue, substrate characterization still heavily relies on bench work methods in heterologous expression systems such as yeast or *Xenopus* oocytes. In this context, the goal of this review is to describe in a simple way the tools that are commonly used to characterize these transporters (Fig. 2) and to provide a reader‐friendly overview of the knowledge concerning the plant NPF. In addition, since the evolution of NPF in plants is unique compared with the other eukaryotic kingdoms and domains of life, this review also delves into the evolution of NPF in land plants and algae (Fig. 4, see later). A relatively extensive Excel file (Table S1), including a wealth of useful information concerning NPF from different species, accompanies this review, providing a ‘starter pack’ for anyone who undertakes to characterize a NPF and would like to find the needle in the haystack.

## II. Characterization methods of NPF transport activity

Heterologous expression is the gold standard to characterize the functional properties of membrane transporters. Various experimental systems provide complementary advantages to investigate transport kinetics, substrate specificity/selectivity, substrate affinity (determined by the *K*_m_ value, defining high affinity if *K*_m_ is below 0.5 mM and low affinity if higher), and physiological roles of transporters. The majority of NPF proteins have been functionally characterized using well‐established heterologous expression systems: *Xenopus laevis* (African clawed frogs) oocytes and yeast (primarily *Saccharomyces cerevisiae*). These two approaches dominate the literature, and their use is detailed below. Fig. 2 provides a schema that can help the reader find the best method to characterize the transport of different substrates in these two heterologous expression systems. Some NPF transporters have been characterized through other heterologous expression approaches/systems, including proteoliposomes from *Lactococcus lactis* (NPF2.3/NAXT2; Taochy *et al*., 2015), insect cell lines (*Sf*9 cells) for NPF4.6/AIT1 (Kanno *et al*., 2012), or *Escherichia coli* (Grunewald *et al*., 2020), which are not detailed in this review.

### 1. Yeast

Identification and characterization of plant membrane transporters have been greatly facilitated by the availability of yeast mutants used for functional complementation and/or transport analysis (Frommer & Ninnemann, 1995; Wittstock *et al*., 2000). The heterologous yeast expression system offers a simplified eukaryotic environment for functional analyses with numerous advantages: ease of genetic manipulation, rapid growth, and cost‐effectiveness, which have enabled efficient screening of NPF transporters through complementation assays and direct substrate uptake measurements.

#### Yeast complementation

To perform functional complementation, appropriate yeast strains deficient in specific transport capabilities (e.g. for dipeptides, K^+^) are transformed with a plasmid carrying the Coding DNA Sequence (CDS) of the studied NPF and a gene that complements the auxotrophy. The ability to restore growth is then tested on selective media. *Saccharomyces cerevisiae* has been particularly instrumental in characterizing NPF transporters involved in peptide transport (predominantly from clade 8), due to the fact that a yeast strain defective in peptide uptake (ΔPTR2) provides a clean background for functional complementation studies and screens for peptide or dipeptide transport by NPF (Rentsch *et al*., 1995; Dietrich *et al*., 2004; Léran *et al*., 2015b). Yeast strain JT16, defective in histidine and arginine permease and in histidine biosynthesis, has also been used to test the ability to transport histidine (Frommer *et al*., 1994).

*Saccharomyces cerevisiae* cannot use nitrate as a nitrogen source. Therefore, the methylotrophic yeast *Ogataea polymorpha* (previously named *Hansenula polymorpha* or *Pichia angusta*) is the only yeast strain known that can assimilate nitrate as its sole nitrogen source and has been used for nitrate transport studies. This nitrate‐dependent nitrogen nutrition involves a unique high‐affinity nitrate transporter YNT1 (Siverio, 2002). The *O. polymorpha* Ynt1 knockout mutant has been functionally complemented by the genes AtNPF6.3/NRT1.1 (Martín *et al*., 2008) and AtNPF6.2/NRT1.4 (Morales de los Ríos *et al*., 2021).

#### Modified two‐hybrid system

Elegant yeast systems derived from the yeast two‐hybrid (Y2H) assay have been crucial in identifying NPF that transport three key hormones, such as ABA, gibberellic acid (GA), and JA and JA‐Ile. These systems leverage the fact that each of these hormones is required to form a protein complex between its receptor (PYR1, GID1a, or COI1 for ABA, GA, or JA, respectively) and a corresponding ‘interactor’ or ‘co‐receptor’ (ABI1, GAI, and JAZ3). The hormone receptors are fused to the GAL4‐DNA binding domain, whereas the interactors are fused to the activation domain. Hormone‐dependent interactions between both proteins are detected by using HIS3 as a selection marker. An NPF‐dependent hormone increase within the yeast induces the expression of HIS3 and allows the yeast to grow in histidine‐free medium (Kanno *et al*., 2012, 2013; Chiba *et al*., 2015).

#### Toxic analogs

Growth inhibition due to the transport of toxic compounds by NPF may provide an alternative selection procedure in yeast. For example, AtNPF2.5‐expressing yeast growth is inhibited by 300 and 400 mM bromate (KBr) (B. Li *et al*., 2017). Bromate has been used as a chloride toxic analog (MacRobbie, 1975) but can also be considered a nitrate analog (Hille, 2001; Dietrich *et al*., 2004). The use of dipeptides with toxic analogs has also been tested, but was only effective for yeast PTR (Song *et al*., 1996).

#### Accumulation studies

Transport and accumulation studies in yeast mutant strains complemented by NPF transporters enabled transport kinetics investigations using radiolabeled peptides and amino acids (mainly [^3^H]‐compounds) by direct measurement of accumulation (or competition experiments) using a liquid scintillation analyzer (Rentsch *et al*., 1995; Dietrich *et al*., 2004). Accumulation studies have also been conducted in wild‐type yeast strains for compounds that are not naturally transported into yeast (such as hormones) following transformation with NPF transporters (Krouk *et al*., 2010; Michniewicz *et al*., 2019).

### 2. *Xenopus* oocytes

Since 1992 (Boorer *et al*., 1992; Schachtman *et al*., 1992), *Xenopus* oocyte is the predominant system for functional characterization of plant transporters due to several key advantages. These large cells (1 mm diameter) provide an ideal environment for heterologous membrane protein expression with minimal endogenous transporter activity and make *Xenopus* oocytes exceptionally well suited for comprehensive functional and kinetic analyses of transporters (Miller & Zhou, 2000). However, this expression system requires oocytes to be surgically extracted from female frogs (Aguero *et al*., 2018), either by purchasing them from commercial suppliers or obtaining them from animal facilities possessing all necessary regulatory approvals and permits.

For proper expression in *Xenopus* oocytes, the complete NPF coding sequence must first be cloned into an oocyte‐compatible expression vector (e.g. pOO2, pGEM‐HE) containing (1) the 5′ and 3′ untranslated regions (UTRs) of *Xenopus* β‐globin (Liman *et al*., 1992), (2) a polyadenylation tail (Gillian‐Daniel *et al*., 1998), and (3) a T7 or SP6 promoter allowing *in vitro* transcription of capped cRNA (Gillian‐Daniel *et al*., 1998). cRNA (1–50 ng) is then injected into each oocyte using precise microinjection techniques (Aguero *et al*., 2018). After injection, oocytes are typically incubated for 2–3 d at 18°C to allow the expression and addressing of NPF transporters at the plasma membrane before conducting functional assays, even for tonoplastic NPFs (Jørgensen *et al*., 2017; Payne *et al*., 2017). Both electrophysiological measurements (using the electrogenicity of the proton‐coupled transport mechanism common in NPF) and substrate accumulation studies with stable, radiolabeled, or fluorescent substrates can be performed the following 2 to 5 d.

#### Accumulation studies

Using NPF‐expressing *Xenopus* oocytes compared with water‐injected controls, accumulation studies have established NPF transporter function by demonstrating significantly modified accumulation of diverse compounds, including nitrate, peptides, GSLs, auxin, ABA, and gibberellic acid. These experiments provide unequivocal evidence of transport activity by measuring substrate accumulation or often by using labeled compounds with radioactive (^3^H, ^37^Cl) or stable (^13^C, ^15^N) isotopes that enable precise quantification of transport rates and kinetic parameters.

For uptake assays, the oocytes are incubated for 15–180 min in a standard bath solution (either ND96, Barth, or MBS Modified Barth Solution, Ringer) in which the tested substrate has been added. Since the accumulation of a tested substrate should be detected in the oocytes within hours if it is indeed the actual substrate of the tested transporter, the interpretation of longer incubation times should be taken with a pinch of salt. The oocytes are then washed five times with cold (4°C) standard bath solution without the substrate or with unlabeled substrate. Substrate accumulation is then measured either in single oocytes or in pools, depending on the technique used for quantification. For stable isotopes‐coupled compounds (^15^N‐nitrate and ^13^C‐glycerate assays), total nitrogen/carbon content and atom percentage ^15^N/^13^C abundance are analyzed by elemental analysis and isotope ratio mass spectrometry (EA‐IRMS; Léran *et al*., 2015a; Lin & Tsay, 2023). For radiolabeled compounds, liquid scintillation analyzers are used (Krouk *et al*., 2010), and for some specific compounds, quantification is performed directly by ultra‐high‐performance liquid chromatography with tandem mass spectrometry (UHPLC/MS‐MS) after supernatant purification (Nour‐Eldin *et al*., 2012; Payne *et al*., 2017).

Efflux experiments can also be performed after direct substrate injection into the oocyte (Léran *et al*., 2013). After incubation, the substrate can be quantified both within the oocyte (where a decrease in substrate concentration would indicate efflux activity) and in the bath solution (where the presence of substrate originating from the oocyte would confirm efflux transport).

Attention should be drawn to membrane diffusion of weak protonated organic acids (such as most phytohormones at acidic pH), which can freely accumulate into the cell, whereas export of these molecules necessitates the activity of a transporter (Boursiac *et al*., 2013). Given that the uptake activity of NPF is often coupled with proton (H^+^) transport, functional tests should be performed in different pH‐buffered solutions. Rapid acidification of the cytoplasm due to H^+^ transport by AtNPF7.3/NRT1.5 has been measured using intracellular (H^+^)‐selective microelectrodes and should be taken into account (Wulff *et al*., 2019; Kanstrup & Nour‐Eldin, 2022) as these intracellular pH changes can modify the charge of weak acids and induce ion‐trap mechanisms (Boursiac *et al*., 2013; Wulff *et al*., 2019).

#### Electrophysiological studies

The two‐electrode voltage clamp (TEVC) method has been performed to demonstrate the electrogenicity of some NPF displaying a proton‐coupled transporter mechanism for nitrate, dipeptides, chloride, GSLs, glucose, sucrose, and GAs (Chiang *et al*., 2004; Dietrich *et al*., 2004; Hammes *et al*., 2010; Nour‐Eldin *et al*., 2012; Wulff *et al*., 2019; Yang *et al*., 2022; Binenbaum *et al*., 2023). The large size of *Xenopus* oocytes allows one to easily use the TEVC technique, which consists of controlling the membrane potential while measuring the current due to the net charge flux across the membrane in real time with two electrodes. Negative currents recorded upon membrane hyperpolarization could be mediated either by the influx of positively charged molecules and/or the efflux of negatively charged molecules (Hille, 2001). For NPF, positively charged molecules can be protons, potassium, or amino acids (Chiang *et al*., 2004; Jørgensen *et al*., 2015), and negatively charged molecules can be chloride, nitrate, hormones, or amino acids (Li *et al*., 2016; B. Li *et al*., 2017). Whatever the NPF tested by TEVC, the generated currents are relatively small, and require careful analysis. Using these approaches, GSLs and sugars cotransporting with protons have been firmly established. The nitrate transport of some NPF, such as NPF6.3, has also been demonstrated to involve a symport activity with protons. The stoichiometry of this symport is not yet firmly established: initial studies suggested a transport of more than one proton per nitrate molecule, transport demonstrated by the recording of negative currents in NPF6.3‐expressing oocytes (Tsay *et al*., 1993; Chiu *et al*., 2004), whereas another study shows that this transporter is nonelectrogenic (Noguero *et al*., 2018). In specific conditions, NPF8.1/PTR1 and NPF8.2/PTR2 can generate leak proton currents, behaving as proton transporters (Hammes *et al*., 2010). Non (or poorly) electrogenic transport of H^+^, K^+^, or chloride for some NPF members has been quantified using the Microelectrode Ion Flux Estimation system (MIFE®)/Non‐invasive Micro‐test System (NMT) technique (B. Li *et al*., 2017; H. Li *et al*., 2017).

Regardless of the substrate tested, endogenous transporters (particularly anion channels) can be present at the plasma membrane of certain oocyte batches and interfere with accurate data analysis, leading to misinterpretation of TEVC results (Terhag *et al*., 2010).

## III. Substrate transport selectivity of NPF

**Fig. 3:**
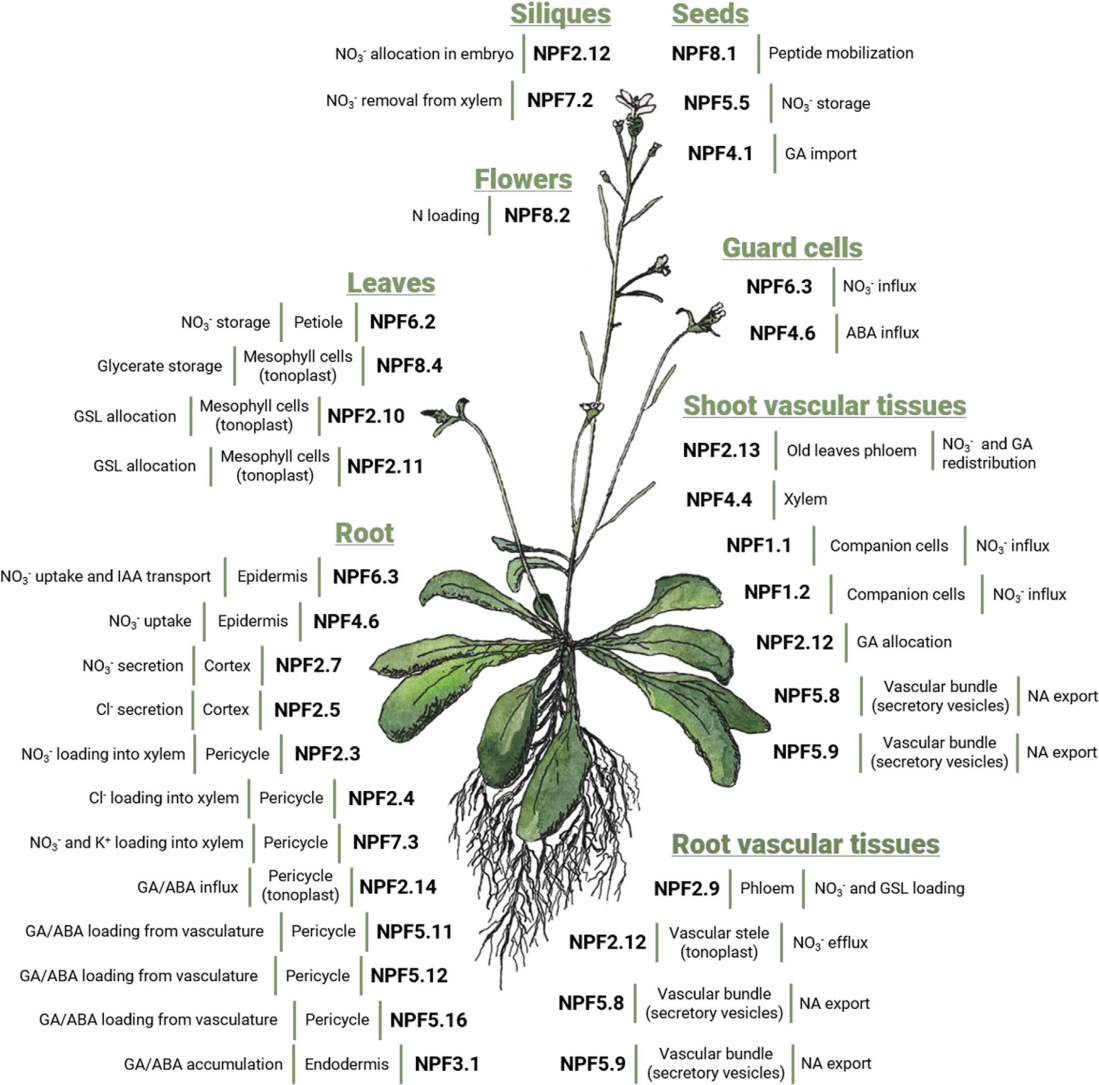
Example of tissue localization and function of some of the main characterized NRT1/PTR FAMILY (NPF) in *Arabidopsis thaliana*. NPFs are located in the plasma membrane unless otherwise indicated. NA, nicotianamine.

The following section is an overview of the current knowledge concerning the different NPF substrates presented by clades. *Arabidopsis thaliana*, a species in which the largest number of NPFs has been characterized (Tables 1, S1 and Fig. 3), is used as a main example, but noteworthy examples from other species are also included (Table S1).

### 1. NPF1

NPF1 consists of three members in Arabidopsis. AtNPF1.1/NRT1.11 and AtNPF1.2/NRT1.12 are both nitrate transporters localized to the plasma membrane of companion cells in leaf veins (Hsu & Tsay, 2013). They are highly expressed in fully expanded leaves and are involved in nitrate loading into the phloem. Although nitrate is not the primary nitrogen form in the phloem, it appears to influence plant growth, as double mutants for *npf1.1/npf1.2* exhibit reduced shoot biomass. Functional characterization in *Xenopus* oocytes has confirmed that both transporters mediate low‐affinity nitrate transport. The AtNPF1.3 transporter was characterized in *Xenopus* oocytes and identified also as a nitrate transporter and has recently been used to design a nitrate biosensor (Chen & Ho, 2022).

In *Medicago truncatula*, the high‐affinity nitrate transport activity of MtNPF1.7/MtNIP/LATD has been demonstrated in *Xenopus* oocytes. When expressed in Arabidopsis, this NPF complements the decreased chlorate sensitivity of the *npf6.3* knockout mutant *chl1‐5*, whereas expression of *AtNPF6.3* in the medicago *npf1.7* KO mutant partially restored the wild‐type phenotype (Bagchi *et al*., 2012), highlighting functional redundancy between different types of NPF.

Using a modified Y2H system (see above), Chiba *et al*. (2015) demonstrated that AtNPF1.1 and AtNPF1.2 also transport GAs (GA1, GA3, and GA4) and jasmonate–isoleucine. Based on these findings, the reduced biomass observed in the *npf1.1/npf1.2* double mutant (Hsu & Tsay, 2013) may not only result from impaired nitrate transport but also from disrupted GA transport.

Interestingly, in tomato (Kazachkova *et al*., 2021), three NPF1 family members (SlNPF1.3, 1.5, and 1.6) have been identified as α‐tomatine transporters. While a monogenic mutation in SlNPF1.5 explains the bitter fruit phenotype observed in all bitter *S. lycopersicum* accessions, the existence of these additional transporters suggests that α‐tomatine transport could also occur in other organs or under specific physiological conditions, highlighting the potential diversity of NPF1 transporter functions. This also demonstrates the potential of NPF in improving fruit quality and shows that these transporters can be involved in the interactions between animals and plants since bitterness is one of the main traits influencing the interactions between frugivore animals and plants.

### 2. NPF2

Since the publication of the NPF nomenclature in 2014 (Léran *et al*., 2014), the NPF2 clade, which consists of 14 members in Arabidopsis, has been divided into two subclades based on sequence homologies and the presence of the ExxER/K motif in NPF2b (Jørgensen *et al*., 2017; Longo *et al*., 2018).

#### NPF2a

In Arabidopsis, NPF2a includes seven members (AtNPF2.1–AtNPF2.7) whose genes are organized as a cluster on chromosome 3. This cluster organization is also often found in other species where this clade is present, indicating a recent duplication of this family and a potential co‐regulation of their expression. AtNPF2.7/NAXT1 was identified as a passive nitrate efflux transporter involved in nitrate secretion from root periphery cells under acidification stress (Segonzac *et al*., 2007) and AtNPF2.3/NAXT2, expressed in root pericycle cells, was found to facilitate nitrate root to shoot translocation under salt stress conditions (Taochy *et al*., 2015). It was suggested that the absence of the ExxER/K motif in NPF2a relates to its capacity to export nitrate (Longo *et al*., 2018).

Two members of this subclade, AtNPF2.4 and 2.5, mediate chloride efflux rather than nitrate efflux. AtNPF2.5 is involved in chloride secretion from the root (B. Li *et al*., 2017) while AtNPF2.4 contributes to root‐to‐shoot transfer by loading chloride into xylem (Li *et al*., 2016) thereby enhancing plant performance upon salt stress.

In addition to their roles in anion transport, Chiba *et al*. (2015) and Wulff *et al*. (2019) revealed that multiple members of this subclade can transport hormones: AtNPF2.1, 2.3/NAXT2, 2.4, 2.5, 2.6, and 2.7/NAXT1 can transport GAs; AtNPF2.4, 2.6, and 2.7 can transport jasmonate–isoleucine; while only NPF2.5 demonstrates the ability to transport ABA within this subclade (Chiba *et al*., 2015; Wulff *et al*., 2019). However, the role of this NPF‐mediated hormone transport remains to be investigated in the context of plant development.

Unexpected secondary metabolite transport from an NPF2a member has been revealed with CrNPF2.9 from *Catharanthus roseus*. Indeed, CrNPF2.9 is a vacuolar exporter of strictosidine, the sole precursor of all monoterpene indole alkaloids (MIA), a class of compounds with important medical properties (e.g. anticancer, antimalarial) and naturally produced by *C. roseus* (Payne *et al*., 2017; Kanstrup & Nour‐Eldin, 2022), highlighting the potential of NPF for medicinal applications.

#### NPF2.b

The clade NPF2.b includes seven members in Arabidopsis (AtNPF2.8–AtNPF2.14) that have predominantly been characterized in *Xenopus* oocytes as H^+^‐dependent nitrate low‐affinity transporters that play crucial roles in nitrate distribution throughout plants. AtNPF2.9/AtNR1.9, which is expressed in root companion cells, facilitates local nitrate redistribution by loading into the phloem. Studies of the *npf2.9* mutant have reported a decreased nitrate content in root phloem exudates and reduced downward nitrate transport (Wang & Tsay, 2011). AtNPF2.12/NRT1.6, expressed in vascular tissues in siliques, plays a significant role in early embryo development. The *npf2.12* mutant exhibits an increased seed abortion rate due to reduced nitrate accumulation (Almagro *et al*., 2008). AtNPF2.13/NRT1.7, expressed in the phloem of mature leaves, is involved in nitrate redistribution from source to sink tissues through phloem loading (Fan *et al*., 2009). In Arabidopsis, AtNPF2.14 is the only member of its subclade for which nitrate transport has not been demonstrated (Binenbaum *et al*., 2023).

In rice, OsNPF2.4 functions as a low‐affinity nitrate transporter involved in nitrate remobilization from source leaves to sink organs and is involved in nitrate uptake from the soil (Xia *et al*., 2015). OsNPF2.2/OsPTR2 expressed in *Xenopus* oocytes is a low‐affinity pH‐dependent nitrate transporter. It plays an important role in the long‐distance transport of nitrate and plant development, with the *Osnpf2.2* mutant displaying abnormal vasculature, retarded plant growth, and impaired seed filling (Li *et al*., 2015).

Transport of GSLs and other secondary metabolites has also been investigated in this NPF subclade. AtNPF2.10/GTR1 and AtNPF2.11/GTR2 have been screened as 4‐methylthiobutyl (4MTB) and indol‐3‐ylmethyl (I3M) transporters using a *Xenopus* oocyte and LC‐MS, and fully characterized using TEVC (Nour‐Eldin *et al*., 2012), whereas AtNPF2.9/GTR3 was demonstrated to be more selective, being able to transport I3M but not 4MTB (Jørgensen *et al*., 2017). These three NPF transporters are high‐affinity H^+^‐dependent GSL transporters, with a *K*_m_ below 30 μM (Nour‐Eldin *et al*., 2012; Jørgensen *et al*., 2017). In addition to natural GSL, these transporters are also able to transport artificial fluorescent GSLs, providing nice tools for furthermore transport studies (Kanstrup *et al*., 2023). They are involved in importing GSLs from the apoplast and thereby have a role in long‐distance GSL transport in the phloem, as evidenced by the *gtr1/gtr2* and *gtr1/gtr2/gtr3* mutants' phenotype, which accumulate root‐born GSLs in the rosette (Nour‐Eldin *et al*., 2012; Andersen *et al*., 2013; Madsen *et al*., 2014; Saito *et al*., 2015; Kanstrup & Nour‐Eldin, 2022). The role of these transporters in seed GSL accumulation has been recently clarified by demonstrating their role in the funiculus, where they load GSL into the phloem (Sanden *et al*., 2024). GSL transporters have been characterized in other plant species: *Brassica juncea*, *Brassica rapa, Carica papaya*, and *Manihot esculenta* (Table S1). MeNPF2.7/CGTR1 from cassava (Manihot esculenta) is involved in the transport of cyanogenic glucosides, toxic compounds that impact food safety (Jørgensen *et al*., 2017; Nambiar *et al*., 2021; Kanstrup & Nour‐Eldin, 2022).

AtNPF2.8/FST1 transports flavonoids deposited within the protective pollen coat (Grunewald *et al*., 2020; Kanstrup & Nour‐Eldin, 2022).

Members of the NPF2b subclade have also been implicated in hormone transport. An initial screen for GA transport using a Y2H system revealed that AtNPF2.10/GTR1, AtNPF2.12/NRT1.6, AtNPF2.13/NRT1.7 are able to transport GAs (Chiba *et al*., 2015). A subsequent screen optimized for oocytes, accounting for passive GA import through diffusion (as they are weakly acidic), refined the list of NPF2b members capable of GA transport to include AtNPF2.9/NRT1.9, AtNPF2.11/GTR2, AtNPF2.12/NRT1.6, and AtNPF2.13/NRT1.7 (Wulff *et al*., 2019). Recently, work from Binenbaum *et al*. (2023) demonstrated that AtNPF2.14, a tonoplast‐localized transporter expressed in the pericycle of the root mature zone, mediates the export of GAs and ABA that accumulate in the root endodermis to facilitate suberization. The *npf2.14* mutant displays significantly stronger accumulation of both hormones, with consequent effects on suberin deposition. AtNPF2.12/NRT1.6 and AtNPF2.13/NRT1.7 were also characterized as GAs and ABA transporters, but function as plasma membrane localized importers that also regulate suberization (Binenbaum *et al*., 2023). AtNPF2.10/GTR1 also weakly imports JA and jasmonate–isoleucine in oocytes, as shown by a long (24 h) transport assay (Saito *et al*., 2015) and has a role in wound responses (Ishimaru *et al*., 2017) and lateral root development through JA signaling (Kuo *et al*., 2020).

### 3. NPF3

The clade NPF3 of *A. thaliana* consists of a single member, AtNPF3.1. Expression in *Xenopus* oocytes and yeast demonstrates that this NPF transports nitrate (Pike *et al*., 2014), nitrite (Sugiura *et al*., 2007; Pike *et al*., 2014) and different types of GAs (David *et al*., 2016; Tal *et al*., 2016). However, *npf3* mutants showed no significant changes in nitrate uptake *in planta* (David *et al*., 2016). *AtNPF3* is expressed in roots, shoot vasculature, and hypocotyl (David *et al*., 2016; Tal *et al*., 2016). AtNPF3.1 imports GAs into endodermal cells of the elongation zone under nitrate starvation (David *et al*., 2016), regulated by GAs, ABA, and salt stress (Tal *et al*., 2016) and is implicated in suberization (Binenbaum *et al*., 2023).

Similarly, the rice ortholog OsNPF3.1 is also capable of transporting nitrate, GAs, and ABA to regulate nitrogen utilization efficiency and tillering (Hang *et al*., 2024). MtNPF3.1 in *Medicago truncatula* and OsNPF3.1 in *Oryza sativa* both retained their GA transport activity at similar levels, indicating functional conservation (Tal *et al*., 2016).

### 4. NPF4

Clade NPF4 is composed of seven members in Arabidopsis. A known property of this clade is ABA transport activity, which has been identified in yeast, insect cells (*Sf*9), or oocytes for AtNPF4.1/AIT3, AtNPF4.2/AIT4, AtNPF4.5/AIT2, AtNPF4.6/NRT1.2, and AtNPF4.7 (Kanno *et al*., 2012; Léran *et al*., 2020). AtNPF4.6/NRT1.2 also exhibited low‐affinity nitrate uptake (Huang *et al*., 1999), and it has been determined that its ability to transport both nitrate and ABA does not reflect any physiological link between these two factors (Kanno *et al*., 2013). However, the dual‐substrate specificity of AtNPF4.6/NRT1.2 is physiologically significant, as it has been shown to function as a constitutive low‐affinity nitrate uptake transporter at the rhizodermis (Huang *et al*., 1999), and as an ABA uptake transporter in leaf guard cells that positively regulates stomatal closure (Shimizu *et al*., 2021). It was shown very recently (Liu *et al*., 2025) that AtNPF4.6 negatively regulates salt tolerance in Arabidopsis. It was suggested that this role *in planta* could be mediated by the capacity of AtNPF4.6 to behave as a pH‐dependent cation (Na^+^, K^+^, Li^+^) importer. However, this result needs to be taken with a pinch of salt since the recorded currents could also have been mediated by a proton/anion (Cl^−^/NO_3_^−^) symporter.

AtNPF4.1 also transports GAs (GA3) in yeast (Kanno *et al*., 2013) and oocytes (Tal *et al*., 2016), with recent findings *in planta* supporting its function as an importer of embryo‐derived GA4 into the endosperm to promote germination (Sirlin‐Josserand *et al*., 2024). AtNPF4.4/NRT1.13 is involved in the control of shoot architecture and flowering under low nitrate conditions (Chen *et al*., 2021a). It is also involved in nitrogen allocation between the different parts of the plant, despite lacking nitrate transport activity due to a single amino acid substitution (proline to serine at 487), suggesting that this NPF might function as a transceptor or as a transporter of an unknown signaling molecule.

In other species, some NPF4 are notable for their unique function, such as OsNPF4.5, a low‐affinity nitrate transporter exclusively expressed in root cells that have been colonized by arbuscular mycorrhiza fungi (AMF), involved in symbiotic nitrogen uptake (S. Wang *et al*., 2020). AMF‐induced expression was also observed with orthologs in maize, sorghum, and Medicago, suggesting the existence of a conserved symbiotic nitrate uptake route facilitated by NPF4.5 at least in grasses (S. Wang *et al*., 2020). In cassava (*Manihot esculenta* Crantz), MeNPF4.5 exhibited dual functionality, acting as a nitrate efflux transporter in seedlings grown on low nitrate medium, and as an influx transporter in seedlings exposed to high concentrations of external nitrate (Zou *et al*., 2023). In *Lotus japonicus*, LjNPF4.6 transports nitrate and ABA, and is involved in promoting lateral root elongation in response to nitrate and ABA stimuli (Alves *et al*., 2024).

### 5. NPF5

Arabidopsis NPF5 clade is composed of 16 members. Among them, AtNPF5.10, AtNPF5.11, AtNPF5.12/TOB1, AtNPF5.14/NRT1.15 and AtNPF5.16 transport nitrate in the low affinity range when tested in *Xenopus* oocytes (He *et al*., 2017; Lu *et al*., 2022). *In planta* they are localized in the tonoplast and have been proposed to mediate nitrate efflux from vacuoles (He *et al*., 2017). AtNPF5.12/TOB1 also transports the auxin precursor indole‐3‐butyric acid (IBA) into the vacuoles (Michniewicz *et al*., 2019), and is involved in defense responses against the pathogen fungi *Verticillium longisporum* (Dölfors *et al*., 2024). Work with *npf5.11/npf5.12/npf5.16* triple mutant shows an effect on nitrate partitioning between roots and shoots (He *et al*., 2017; Lu *et al*., 2022). AtNPF5.5 is also involved in nitrate transport and is localized in the embryo, where it mediates nitrogen storage (Léran *et al*., 2015b). In rice, OsNPF5.16 has been characterized as a low affinity nitrate transporter in *Xenopus* oocytes. This transporter is predominantly located in epidermal and vascular cells in roots, and experiments with overexpressing and RNA interference lines in the *japonica* rice variety ZH11 show its role in promoting tillering and grain filling (Wang *et al*., 2022).

Abscisic acid, GAs, and/or jasmonate–isoleucine uptake was reported for AtNPF5.1, AtNPF5.2/PTR3, AtNPF5.3, AtNPF5.6, AtNPF5.7 using a yeast heterologous expression system (Chiba *et al*., 2015). *In planta*, *AtNPF5.1* is expressed in vascular tissues, leaf mesophyll, epidermal cells during vegetative stages and also in the seed coat at late stages of seed development. Its expression is induced by exogenous ABA treatment, and the *npf5.1 ko* mutant exhibits a higher leaf surface temperature than in the wild‐type, suggesting a role in stomatal closure (Shimizu *et al*., 2021, 2022). In addition to ABA and GAs, AtNPF5.2/PTR3 is able to transport dipeptides, and its expression is induced by biotic and abiotic stresses in roots, leaves and seeds (Karim *et al*., 2005, 2007). The *AtNPF5.2* gene is required for defense to limit the infection by virulent *Pseudomonas*, and its regulation is likely to be influenced by both JA and salicylic acid pathways (Karim *et al*., 2007).

In addition to transporting nitrate, AtNPF5.8/NAET1 and AtNPF5.9/NAET2 also transport NA (a vitamin B3 form). The transport of nitrate and NA is independent and has no influence on each other (Chen *et al*., 2021b). NA is required to transport iron into the shoot in order to prevent its precipitation in the vascular tissues (Stephan *et al*., 1996), suggesting a role for AtNPF5.8/NAET1 and AtNPF5.9/NAET2 in iron transport. AtNPF5.8/NAET1 and AtNPF5.9/NAET2 are proposed to transport cytoplasmic NA into secretory vesicles, which then secrete NA by exocytosis into the apoplastic space, where it encounters free metal ions or exchanges metals with other metal chelates to form the metal‐NA complexes (Chao *et al*., 2021). *AtNPF5.8* and *AtNPF5.9* are predominantly expressed in vascular tissues and are highly upregulated in response to Fe^2+^ deficiency. Double mutants showed severe defects in seed germination (Chen *et al*., 2021b).

### 6. NPF6

Clade NPF6 consists of four members in Arabidopsis. AtNPF6.3/NRT1.1/CHL1, which is the first plant NPF characterized as a nitrate and auxin transporter, is by far the most‐studied NPF in plants (Tsay *et al*., 1993; Wang *et al*., 1998; Huang *et al*., 1999; Liu & Tsay, 2003; Krouk *et al*., 2010). It is also the first NPF for which a signaling activity has been reported (Muños *et al*., 2004), getting this protein to be regularly referred to as a transceptor (Ho *et al*., 2009).

In *Xenopus* oocytes, nitrate transport activity of AtNPF6.3/NRT1.1 is bidirectional (Léran *et al*., 2013) and influx has been proposed to behave at both high and low affinities (Wang *et al*., 1998; Liu & Tsay, 2003; Ho *et al*., 2009), an unusual property shared with MtNPF6.8 (Morère‐Le Paven *et al*., 2011; Pellizzaro *et al*., 2014). It is thought that AtNPF6.3/NRT1.1 switches between these two affinities by CIPK23‐dependent phosphorylation of T101 (Ho *et al*., 2009). According to the proposed model, under high nitrate availability (> 1 mM), AtNPF6.3/NRT1.1 forms a homodimer, which behaves as a low‐affinity transporter. When the external nitrate concentration drops below 1 mM, phosphorylation of AtNPF6.3^T101^ by CIPK23 switches its transport to high affinity, which might occur through decoupling of AtNPF6.3/NRT1.1 dimers (Sun & Zheng, 2015). However, the affinity shift of AtNPF6.3/NRT1.1 is controversial because AtNPF6.3^T101^ phosphorylation does not change *K*_D_ value for nitrate binding (Parker & Newstead, 2014) and because the capacity of AtNPF6.3/NRT1.1 to transport nitrate in a high‐affinity range is strongly dependent on the experimental setup (Noguero *et al*., 2018). The proposed double affinity could be due to the fact that in experiments carried out in heterologous expression systems, the rates of nitrate uptake obtained were fitted using two separate Michaelis–Menten curves, resulting in two *K*_m_ values for high‐ and low‐affinity activities (*K*_m_ ≈ 50 μM and ≈ 4 mM, respectively) (Liu *et al*., 1999; Liu & Tsay, 2003). However, the application of a single Michaelis–Menten equation to the published data provided the best fit (Martín *et al*., 2008; Glass & Kotur, 2013; Wen & Kaiser, 2018; Ye *et al*., 2019). In addition, *in planta* influx experiments failed to show high‐affinity transport (Muños *et al*., 2004). Nitrate transport activity was also detected for ZmNPF6.6 (Wen *et al*., 2017) and OsNPF6.5 (Hu *et al*., 2015) in oocytes at low external concentrations (200 μM nitrate). In this context, it is important to consider that nitrate transport occurring at low external nitrate concentration does not directly correlate with high‐affinity transport.

The structural analysis of AtNPF6.3/NRT1.1 suggests a dimeric configuration comprising two nearly identical protomers. As mentioned before, the docking and undocking dynamics of this dimer are hypothesized to be regulated through the phosphorylation of this T101 residue (Ho *et al*., 2009; Parker & Newstead, 2014; Sun *et al*., 2014). AtNPF6.3^T101^ residue is highly conserved among NPF6 members, and its phosphorylation seems to have different effects depending on the NPF transporter and/or substrate analyzed. In other NPFs, the phosphorylation of AtNPF6.2^T98^ and MtNPF6.5^T101^ reduces the nitrate uptake in *Xenopus* oocytes (Morales de los Ríos *et al*., 2021; Xiao *et al*., 2021). In *Zea mays*, the high‐affinity chloride transport carried out by ZmNPF6.4 was converted into low affinity by ZmNPF6.4^T106A^ and ZmNPF6.4^T106D^ mutations, whereas ZmNPF6.6^T104A^ and ZmNPF6.6^T104D^ both reduce the *K*_m_ of the nitrate transport (Wen *et al*., 2017). Other critical motifs related to nitrate transport by AtNPF6.3/NRT1.1 have also been identified, including H356 and a salt bridge predicted between K164 and E476 (crucial for nitrate binding), as well as P492 (associated with plasma membrane localization and transport function) (Bouguyon *et al*., 2015). However, caution is needed when generalizing discoveries concerning AtNPF6.3/NRT1.1, as other NPF transporters that lack these specific residues yet remain able to transport nitrate.

*In planta*, AtNPF6.3/NRT1.1 is expressed both in shoot and root tissues. AtNPF6.3/NRT1.1 expression in guard cells promotes nitrate‐dependent stomata opening in light conditions (Guo *et al*., 2001; Kou *et al*., 2025). In roots, AtNPF6.3/NRT1.1 facilitates nitrate uptake and root‐to‐shoot translocation (Huang *et al*., 1999; Léran *et al*., 2013), and its auxin (particularly for 2,4‐D and IAA) transport capacity controls root development in response to nitrate availability in the soil (Krouk *et al*., 2010; X. Zhang *et al*., 2019). When the nitrate concentration in the medium is low, AtNPF6.3/NRT1.1 mediates auxin efflux from apical cells of lateral roots, reducing auxin concentration in the apex, which inhibits root elongation. Conversely, the presence of nitrate in the medium decreases AtNPF6.3/NRT1.1 auxin transport capability. Thus, auxin accumulates at the tip of the lateral root, stimulating its elongation (Krouk *et al*., 2010). AtNPF6.3/NRT1.1 thereby connects nutrient availability to hormonal control of root development.

The role of AtNPF6.3/NRT1.1 in nitrate signaling goes even further (Y.‐Y. Wang *et al*., 2018; W. Wang *et al*., 2020; Maghiaoui *et al*., 2020). AtNPF6.3/NRT1.1 is implicated in the rapid expression of genes related to nitrate transport and assimilation, also known as primary nitrate response (PNR) (Muños *et al*., 2004; Bouguyon *et al*., 2015). During this regulation, AtNPF6.3/NRT1.1 is required to produce the transient increase in calcium in response to nitrate (Riveras *et al*., 2015), necessary for the activation of the nitrate–Ca^2+^–dependent protein kinase (CPK) NIN‐LIKE PROTEIN 7 (NLP7) signaling pathway that leads to the nuclear sequestration of NLP7, a transcription factor required for the transcription of PNR genes (B. Li *et al*., 2017; H. Li *et al*., 2017). *MtNPF6.8* and *OsNPF6.5/OsNRT1.1b* also play a role in nitrate signaling. *MtNPF6.8* regulates the expression of *NR* genes either at low and high nitrate conditions (Pellizzaro *et al*., 2014). *OsNPF6.5/OsNRT1.1b* has also been demonstrated to sense external nitrate, and physically interact with *OsSPX4*, linking nitrate and phosphate signaling pathways (W. Wang *et al*., 2020). These moonlighting functions demonstrate the pivotal role of these NPF6 in nitrate sensing and signaling.

Amazingly, the role of NPF6 as a receptor may not be limited to nitrate. Indeed, OsNPF6.5/NRT1.1B was recently demonstrated to be an ABA receptor involved in the crosstalk response to low nitrate and ABA during drought response (Ma *et al*., 2025). It was shown that ABA directly promotes the interaction between OsNPF6.5/NRT1.1B with OsSPX4, thereby releasing the transcription factor NLP4 involved in the transcriptional response to nitrate and ABA (Ma *et al*., 2025). This mechanism seems to be conserved in Arabidopsis and wheat. Hence, OsNPF6.5/NRT1.1B mediates the previously reported antagonistic effect between nitrate and ABA signaling (Ma *et al*., 2025), in a manner similar to what was reported for nitrate and auxin through AtNRT1.1 (Krouk *et al*., 2010). This clearly demonstrates that NPF6 plays a pivotal role in integrating nutrient and hormone signals in order to coordinate metabolic and developmental processes in response to multiple environmental cues.

AtNPF6.2/NRT1.4, the other characterized member of the Arabidopsis NPF6 clade, transports nitrate at a low‐affinity range, and its gene is expressed in the leaf vasculature, where it is involved in nitrate homeostasis by regulating nitrate storage in the petiole (Chiu *et al*., 2004; Morales de los Ríos *et al*., 2021). Nitrate transport has been demonstrated in NPF6 members such as SaNPF6.3, ZosmaNRT1.1, MtNPF6.5, MtNPF6.7, OsNPF6.3, OsNPF6.5, ZmNPF6.4 and ZmNPF6.6 (Fan *et al*., 2016; W. Wang *et al*., 2018; Xiao *et al*., 2021; Nedelyaeva *et al*., 2023; Rubio *et al*., 2023; Cao *et al*., 2024), highlighting the importance of the NPF6 clade in nitrate transport.

The capacity of AtNPF6.3/NRT1.1, ZmNPF6.6, ZmNPF6.4, and MtNPF6.5 to transport chloride has also been demonstrated in *Xenopus* oocytes (Wen *et al*., 2017; Xiao *et al*., 2021). In relation to this, in *Nicotiana tabacum*, the KO mutant of *NtNPF6.13* shows a reduced chloride content in the roots, supporting its participation in chloride uptake (H. Zhang *et al*., 2022).

### 7. NPF7

Clade NPF7 of Arabidopsis consists of three plasma membrane transporters. *AtNPF7.1* is expressed mainly in the anther and pollen. Its expression is upregulated under nitrogen starvation, and its loss resulted in decreased organic nitrogen export from leaves (Babst *et al*., 2019). AtNPF7.2/NRT1.8 is a low‐affinity nitrate transporter expressed mainly in xylem parenchyma cells from root to shoot, as well as flowers and siliques (Tsay *et al*., 2007; Li *et al*., 2010). It is involved in removing nitrate from the ascending xylem sap to avoid build‐up in the shoot under heavy metal stress (Li *et al*., 2010).

The closely related AtNPF7.3/NRT1.5 is a low‐affinity bidirectional nitrate transporter expressed in root pericycle cells, where it loads nitrate into nearby xylem for long‐distance transport (Lin *et al*., 2008). It is likely that AtNPF7.3/NRT1.5 works together with AtNPF7.2/NRT1.8 to fine‐tune nitrate distribution, supported by oppositely regulated expression in the roots (Li *et al*., 2010). Xylem sap K^+^ levels measured in knockout mutants indicate that AtNPF7.3/NRT1.5 is involved in the root‐to‐shoot translocation of this ion to promote accumulation in the foliar organs, which helps suppress leaf senescence induced by nitrate starvation (Meng *et al*., 2016; Zheng *et al*., 2016). This NPF is proposed to act as a relay, transducing nitrate starvation‐derived signals to prominent K^+^ transporters such as HAK5, AKT1, as well as their respective upstream regulators (RAP2.11, ANN1) (Meng *et al*., 2016). Currently, it has been determined that AtNPF7.3/NRT1.5 functions synergistically with the highly selective channel AtSKOR (Drechsler *et al*., 2015), responsible for loading potassium into root xylem sap (Gaymard *et al*., 1998). Whether AtNPF7.3/NRT1.5 is truly capable of *in planta* K^+^ transport or not is unresolved. Although results from *Xenopus* oocytes and yeast indicated K^+^ efflux activity coupled to H^+^ influx (Li *et al*., 2017b), conclusive *in planta* evidence does not exist currently, and it was assumed that due to AtNPF7.3/NRT1.5 localization on root parenchyma cells (exposed to xylem sap with pH 5.5–6.5), K^+^ efflux activity must occur as a result (Lin *et al*., 2008). The tomato SlNPF7.7/NRT1.5 mediates potassium efflux in *Xenopus* oocytes, and its contribution to potassium translocation in plants (together with SlSKOR) under low pH and low K/N supply has been published (Martínez‐Martínez *et al*., 2024). The maize NPF7.9 (also known as SUGCAR1) and NPF7.10 are also able to transport nitrate and potassium in heterologous expression systems (Yang *et al*., 2022; Hu *et al*., 2025). With potassium, proton, and nitrate, the other substrates of this transporter are sucrose and glucose, which are cotransported with protons, suggesting a role in the interactions between nitrogen and carbon fluxes. Sugars and proton fluxes are not affected by potassium, but sugar transport is affected by nitrate (Yang *et al*., 2022).

It is worth noting that AtNPF7.3/NRT1.5 is able to significantly import H^+^ upon exposure to low external pH, which affects diffusion‐based accumulation of hormones (Wulff *et al*., 2019). This does not, however, equate to a complete lack of phytohormone transport activity, as direct transport assays in yeast have shown that AtNPF7.3/NRT1.5 is capable of importing auxin and its precursor IBA, with higher specificity for the latter (Watanabe *et al*., 2020). This was further supported by defective root gravitropism along with reduced IBA levels and auxin responses in knockout mutants (Watanabe *et al*., 2020) and supports the hypothesis that AtNPF7.3/NRT1.5 could be involved in auxin homeostasis in lateral roots under K^+^ starvation (Zheng *et al*., 2016).

Rice NPF7s are 11 members exhibiting very similar roles, some of which have been characterized as nitrate influx transporters in heterologous expression systems, localized at the plasma membrane and tonoplast (Fan *et al*., 2014; Huang *et al*., 2018, 2019; J. Wang *et al*., 2018; Guan *et al*., 2022; M. Zhang *et al*., 2022). In terms of function, 6 of the 11 members have been characterized as regulators of tillering, pinpointing the potential of NPF in crop improvement (Table S1).

### 8. NPF8

The NPF8 clade in Arabidopsis consists of five members, all involved in the transport of amino acids, dipeptides, and tripeptides (longer peptides have been tested but are not transported), though they participate in distinct physiological processes. This clade was previously referred to as PTR due to the structural and functional similarities with the PTR from yeast. Like yeast PTR, members of the NPF8 clade are mainly involved in peptide transport. AtNPF8.1/PTR1 and AtNPF8.2/PTR5 are di‐ and tripeptide transporters. AtNPF8.1/PTR1 plays a role in mobilizing peptides from seed storage reserves to support seedling growth (Dietrich *et al*., 2004; Komarova *et al*., 2012). At later developmental stages, its vascular localization suggests a function in long‐distance transport. Similarly, AtNPF8.2/PTR5 is primarily involved in loading nitrogen compounds into reproductive organs (Hammes *et al*., 2010). AtNPF8.3/PTR2/NTR1/PTR2B was initially described as a histidine transporter (Frommer *et al*., 1994) but later this was not confirmed. Nevertheless, its capacity to transport di‐ and tripeptides has been confirmed in yeast and oocytes (Song *et al*., 1996; Chiang *et al*., 2004). It is essential for pollen grain formation, and the *npf8.2* mutant exhibits delayed flowering (Song *et al*., 1996; Choi *et al*., 2020), indicating a role in floral transition. More recently, AtNPF8.4/PTR4 has been characterized through heterologous expression and *in planta* studies, confirming its role as a glycerate transporter (Weichert *et al*., 2012; Lin & Tsay, 2023). *AtNPF8.4* exists in three transcript variants of different lengths, each displaying varying affinities for glycerate. Finally, AtNPF8.5/PTR6 is involved in the transport of amino acids from the vacuole to the cytosol, particularly during leaf senescence and pollen tube growth, where it supplies metabolites essential for cellular metabolism (Weichert *et al*., 2012; Lu *et al*., 2022). Another sequence (PTR2A) was mistakenly published as a plant peptide transporter (Steiner *et al*., 1994) but was later shown to be a yeast peptide transporter (Steiner *et al*., 2000).

In rice, OsNPF8.1/OsPTR7 is a dimethylarsenate transporter, a property shared with AtNPF8.1 and AtNPF8.2, and is involved in long‐distance translocation of dimethylarsenate and its accumulation in the grain (Tang *et al*., 2017).

## IV. Evolution of NPF in plants

As mentioned in the introduction, NPFs are not specific to plants and belong to a family of proton‐coupled oligopeptide transporters (POT) found in all domains of life. In humans, these transporters belong to the SLC15 family and contribute, among other things, to the intestinal and kidney absorption of di‐ or tripeptides required for nitrogen nutrition (Viennois *et al*., 2018). In yeast, their orthologues are known as PTRs and are also involved in the transport of peptides across membranes (Ito *et al*., 2013). This peptide transport activity is also found in POT homologues found in archaea and bacteria (Harder *et al*., 2008; von Wittgenstein *et al*., 2014). This suggests that POT/PTR/NPF‐related proteins found in the three domains of life might have evolved from an ancestral peptide transporter present in the last universal common ancestor (LUCA). LUCA is likely to have evolved in aqueous environments containing basic organic molecules, including certain amino acids and small peptides that could support its primitive metabolism, as it was unable to synthesize all the organic molecules necessary to survive. Being able to import such organic molecules through transporters like PTR‐related proteins must have undoubtedly conferred a critical asset for early life to survive.

Compared with other kingdoms and domains of life, plant NPF have greatly diversified in number and functions, especially in land plants. A study focusing on GSL transport in Brassicales highlighted that the substrate specificity of different members of the clade 2 NPF accompanies the evolution of new substrates in a short evolutionary period (Jørgensen *et al*., 2017). This shows that the substrate specificity of NPF can evolve very quickly, explaining their great diversity of substrates reported in flowering plants. This also makes it difficult to infer the role of an uncharacterized NPF based on sequence homology with characterized NPF from other species.

**Fig. 4:**
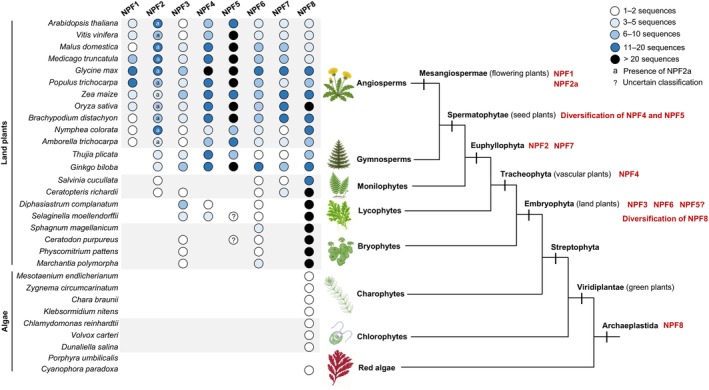
Number of NRT1/PTR FAMILY (NPF) homologs found in different species of red algae and viridiae plants. The homologs have been classified based on a BLASTP against Arabidopsis using the classification established by Léran *et al*. (2014).

The diversity of the roles of NPF among plant species, combined with the lack of characterization of NPF in diverse groups of plants, makes it difficult to draw strong conclusions concerning the roles of NPF in plant evolution. However, comparing the diversification of NPF among evolutionary distant species enables us to speculate concerning their evolution and provides hints to improve our understanding of their roles in the physiology and development of extant species (Fichtner *et al*., 2021). Fig. 4 shows the number of NPF homologues found in different monophyletic groups of plants and classified into the eight clades of NPF determined in Léran *et al*. (2014), based on BLAST analysis against the Arabidopsis proteome. This comparative analysis shows that these eight clades of NPF are found across angiosperms (flowering plants), while gymnosperms only possess seven NPF clades, clade 1 being the missing one. In addition, while NPF2a is found in all tested angiosperm species, the tested gymnosperms only possess NPF2b, suggesting that NPF2a has also evolved in angiosperms. Only one NPF1 and one NPF2a are found in *Amborella trichopoda*, the most distant flowering plant to all other species of this group, further supporting that these types of NPF have evolved in a common ancestor of flowering plants. In tomatoes, NPF1.5 is involved in the molecular mechanism that makes fruits attractive to frugivorous animals (Kazachkova *et al*., 2021). One could speculate that this clade of NPFs has played a role in the seed dispersal of flowering plants via attracting animals. NPF1 and NPF2a clades were reported to transport GAs, a class of hormones that is suspected to have evolved in vascular plants (Hernández‐García *et al*., 2021). GAs play multiple roles in plant development and in the integration of environmental stimuli (Hernández‐García *et al*., 2021). One noticeable role of GAs is their role in floral induction (Yu *et al*., 2012). It is tempting to speculate that the evolution of NPF1 and NPF2a in flowering plants might have played a role in the control of sexual reproduction by the environment, hence providing adaptability.

The NPF5 is usually the largest clade in seed plants, while it is usually absent in other groups of plants. In Arabidopsis and rice, NPF from this clade was reported to play a role in seed development and grain filling, suggesting that the NPF5 clade could have played a role in the evolution of this developmental feature in spermatophytes. This clade of NPF can also transport a broad range of substrates, including different hormones that play roles in developmental plasticity and defense against pathogens, which could have been particularly useful for plants to adapt and survive in harsh conditions.

Monilophytes (ferns), lycophytes, and bryophytes only possess three to five NPF clades. In these groups of land plants, NPF related to the NPF8 group is much more numerous than in the other clades, even when compared with clade eight from seed plants. Strikingly, algal species only possess one or two NPF that are related to the NPF8 clade based on protein sequence similarities, and are sometimes absent, as observed in red algae. This indicates that the diversity of NPF clades in land plants has evolved from an ancestral NPF8‐related gene found in the common ancestor of plants. Furthermore, the massive diversification of NPF in land plants suggests an important role of these transporters during the terrestrialization event. NPF8 proteins are mostly peptide transporters. The over‐representation of the members of this clade in cryptogamic land plants clearly indicates an important role of peptide transport in these species. Given the total lack of NPF characterized in seedless land plants, this role remains unknown. However, it could be speculated that this diversification of peptide transporters might have played a role in the spatial redistribution of nitrogen resources between plant tissues that are more complex in land plants than in algae. In addition, while an aqueous environment enables all parts of algae to access nitrogen resources in their surroundings, on land, nitrogen resources are compartmented and mostly present in the soil. Improvement of nitrogen resource redistribution would have undoubtedly been an advantage for land colonization by plants 450 million years ago.

It was speculated that NPF transports nitrate in algae (Sanz‐Luque *et al*., 2015). However, the transport activity of algal NPF has only been tested in diatoms (*Phaeodactylum tricornutum*) and, despite structural evidence suggesting that PtNPF1 could transport nitrate, no nitrate transport activity was detected in *Xenopus* oocytes so far (Santin *et al*., 2024). This result is not necessarily surprising given that, based on protein sequence comparison, PtNPF1 seems to belong to the NPF8 clade, which mostly contains peptide transporters. More work is urgently needed to characterize NPF in groups of land plants other than flowering plants and algae to unravel the mystery of the role of NPF in plant evolution. This is especially needed to understand why, after the terrestrialization event, NPF has greatly diversified in number and substrate affinity compared with other eukaryotic kingdoms and domains of life.

## V. Conclusion, recommendations, and perspectives

When the first NPF was cloned and characterized as a nitrate transporter, nobody could have imagined that, more than 30 years later, so many different substrates would have been identified. Until 1993, it was thought that proteins sharing sequence and structural homology with PepT/PTR/POT transporters functioned as H^+^/(di/tri)peptides symporters. The cloning of AtNRT1.1/CHL1, later named AtNPF6.3, first demonstrated to be a nitrate transporter (Tsay *et al*., 1993) and then an auxin transporter (Krouk *et al*., 2010), revolutionized our thinking about the structure–function relationships of this superfamily. Even if the function of this unique transporter is a matter for discussion (Glass & Kotur, 2013; Noguero *et al*., 2018; Wen & Kaiser, 2018), the central role of this protein in nitrate transport and sensing is definitively proved (Ruffel *et al*., 2025). The recently demonstrated role of OsNPF6.5/NRT1.1B as an ABA receptor (Ma *et al*., 2025) is adding a new exciting role for this family and clearly shows that NPF plays more than one role in plant nutrition and nitrate signaling. Nevertheless, there is still a long way to go before we establish a full list of NPF substrates and signaling functions.

Identifying the substrate from the protein sequence is not yet possible, but we hope that, for the next NPF review, *in silico*‐based approaches will provide us the opportunity to infer the substrate(s) of each NPF. In the meantime, this review will provide valuable assistance for the search for NPF substrates, based on their phylogenetic organization into eight clades. When trying to identify the substrate of an uncharacterized NPF, besides the use of phenotypes, gene regulation, tissue‐specific expression, you can start your screening with the following molecules depending on the clade: for an NPF from clade 1, 2a, 3, or 5, you should primarily test nitrate or GAs as candidate substrates; for an NPF2b, nitrate and GSLs should be tested; for an NPF4, ABA should be tested; for an NPF6, nitrate, chloride and auxin should be tested; and for an NPF7, nitrate (possibly H^+^/K^+^); and for an NPF8, di‐ and tripeptides (glycerate) should be tested first. But, you should not forget that other specific substrate(s) are likely to be expected, and in this case, it will be like finding a pearl in a pile of oyster shells.

The choice of the expression system for substrate identification will mainly be made between yeast and *Xenopus* oocytes (even if other expression systems were appropriately used in some specific cases). Yeast will be mainly used as a screening system for peptide screening using functional complementation, whereas *Xenopus* oocytes will be used in most other instances. Transport activity will be assessed through LC‐MS quantification, the accumulation of labeled molecules, and/or the two‐electrode voltage clamp technique. Because most NPF substrates are uncharged molecules, electrophysiological approaches provide only indirect evidence of transport, and the recorded currents result from cotransported ions, mainly protons. Accumulation assays should therefore complement these measurements. If feasible, quantification of the unlabeled substrates is the definitive proof of transport. However, labeled molecules are most often used, which is best if the incubation time is relatively short (< 3 h) and the substrates are not metabolized.

Effort is also needed to fully understand how NPFs transport their substrate(s). Indeed, for most NPF, the cotransport mechanisms and stoichiometry underpinning their activity are not well elucidated, except for GSL transporters (NPF2b) and di/tripeptide transporters (NPF8), which are proton‐coupled symporters (Jørgensen *et al*., 2017). To achieve this, electrophysiological approaches in *Xenopus* oocytes combined with structural data should be used to unravel the stoichiometry of the other transporters.

Be that as it may, the plant NTR1/PTR Family stands out from nonplant organisms by its tremendous diversity of substrates and moonlighting functions. This places NPF in a central position in the integration of multiple signaling pathways and their interactions with plant nutrition, thereby enabling plants to adapt their physiology and development to their environment. However, work in more diverse species is needed to fully understand the role of this mesmerizing family in land plant evolution.

## Competing interests

None declared.

## Disclaimer

The New Phytologist Foundation remains neutral with regard to jurisdictional claims in maps and in any institutional affiliations.

## Supporting information


**Table S1** NPF in *Arabidopsis thaliana* (expanded) and in other plant species.
Please note: Wiley is not responsible for the content or functionality of any Supporting Information supplied by the authors. Any queries (other than missing material) should be directed to the *New Phytologist* Central Office.
